# Visual SLAM: What Are the Current Trends and What to Expect?

**DOI:** 10.3390/s22239297

**Published:** 2022-11-29

**Authors:** Ali Tourani, Hriday Bavle, Jose Luis Sanchez-Lopez, Holger Voos

**Affiliations:** 1 Interdisciplinary Centre for Security, Reliability and Trust (SnT), University of Luxembourg, 1855 Luxembourg, Luxembourg; 2 Faculty of Science, Technology and Medicine (FSTM), Department of Engineering, University of Luxembourg, 1359 Luxembourg, Luxembourg

**Keywords:** visual SLAM, Computer Vision, robotics

## Abstract

In recent years, Simultaneous Localization and Mapping (SLAM) systems have shown significant performance, accuracy, and efficiency gain. In this regard, Visual Simultaneous Localization and Mapping (VSLAM) methods refer to the SLAM approaches that employ cameras for pose estimation and map reconstruction and are preferred over Light Detection And Ranging (LiDAR)-based methods due to their lighter weight, lower acquisition costs, and richer environment representation. Hence, several VSLAM approaches have evolved using different camera types (e.g., monocular or stereo), and have been tested on various datasets (e.g., Technische Universität München (TUM) RGB-D or European Robotics Challenge (EuRoC)) and in different conditions (i.e., indoors and outdoors), and employ multiple methodologies to have a better understanding of their surroundings. The mentioned variations have made this topic popular for researchers and have resulted in various methods. In this regard, the primary intent of this paper is to assimilate the wide range of works in VSLAM and present their recent advances, along with discussing the existing challenges and trends. This survey is worthwhile to give a big picture of the current focuses in robotics and VSLAM fields based on the concentrated resolutions and objectives of the state-of-the-art. This paper provides an in-depth literature survey of fifty impactful articles published in the VSLAMs domain. The mentioned manuscripts have been classified by different characteristics, including the novelty domain, objectives, employed algorithms, and semantic level. The paper also discusses the current trends and contemporary directions of VSLAM techniques that may help researchers investigate them.

## 1. Introduction

Simultaneous Localization and Mapping (SLAM) refers to the process of estimating an unknown environment’s map while monitoring the location of an *agent* at the same time [1]. Here, the *agent* can be a domestic robot [2], an autonomous vehicle [3], a planetary rover [4], even an Unmanned Aerial Vehicle (UAV) [5,6] or an Unmanned Ground Vehicle (UGV) [7]. In situations where a prior map of the environment is unavailable, or the robot’s location is unknown, SLAM can be utilized to cover a wide range of applications. In this regard, and considering the ever-growing applications of robotics, SLAM has gained huge attention among industry and research community members in recent years [8,9].

SLAM systems may use various sensors to collect data from the environment, including Light Detection And Ranging (LiDAR)-based, acoustic, and vision sensors [10]. The vision sensors category covers any variety of visual data detectors, including monocular, stereo, event-based, omnidirectional, and Red Green Blue-Depth (RGB-D) cameras. A robot equipped with a vision sensor uses the visual data provided by cameras to estimate the position and orientation of the robot with respect to its surroundings [11]. The process of using vision sensors to perform SLAM is particularly called Visual Simultaneous Localization and Mapping (VSLAM). Utilizing visual data in SLAM applications has the advantages of cheaper hardware requirements, more straightforward object detection and tracking, and the ability to provide rich visual and semantic information [12]. The captured images (or video frames) can also be used for vision-based applications, including semantic segmentation and object detection, as they store a wealth of data for processing. The mentioned characteristics have recently made VSLAM a trending topic in robotics and prompted robotics and Computer Vision (CV) experts to perform considerable studies and investigations in the last decades. Consequently, VSLAM can be found in various types of applications where it is essential to reconstruct the 3D model of the environment, such as autonomous, Augmented Reality (AR), and service robots [13].

As a general benchmark introduced by [14] to tackle high computational cost, SLAM approaches mainly contains two introductory threads to be executed in parallel, known as *tracking* and *mapping*. Hereby, a fundamental classification of the algorithms used in VSLAM is how researchers employ distinct methods and strategies in each thread. The mentioned solutions look differently at SLAM systems based on the type of data they use, making them dividable into two categories: *direct* and *indirect (feature-based)* methods [15]. *Indirect* methods extract feature points (i.e., keypoints) obtained from textures by processing the scene and keeping track of them by matching their descriptors in sequential frames. Despite the computationally expensive performance of feature extraction and matching stages, these methods are precise and robust against photometric changes in frame intensities. *Direct* algorithms, on the other hand, estimate camera motions directly from pixel-level data and build an optimization problem to minimize the photo-metric error. By relying on photogrammetry, these methods utilize all camera output pixels and track their replacement in sequential frames regarding their constrained aspects, such as brightness and color. These characteristics enable *direct* approaches to model more information from images than *indirect* techniques and enable a higher-accuracy 3D reconstruction. However, while direct methods work better in texture-less environments and do not require more computation for feature extraction, they often face large-scale optimization problems [16], and various lighting conditions negatively impact their accuracy. The pros and cons of each approach encouraged researchers to think about developing *hybrid* solutions, where a combination of both approaches is considered. *Hybrid* methods commonly integrate the detection stage of *indirect* and *direct*, in which one initializes and corrects the other.

Additionally, as VSLAMs mainly include a Visual Odometry (VO) front-end to locally estimate the path of the camera and a SLAM back-end to optimize the created map, the variety of modules used in each category results in implementation variations. VO provides a preliminary estimation of the location and poses of the robot based on local consistencies, which are sent to the back-end for optimization. Thus, the primary distinction between VSLAM and VO is whether or not to take into account the global consistency of the map and the predicted trajectory. Several state-of-the-art VSLAM applications also include two additional modules: *loop closure detection* and *mapping* [15]. They are responsible for recognizing previously visited locations for more precise tracking and map reconstruction based on the camera pose.

**Figure 1:**
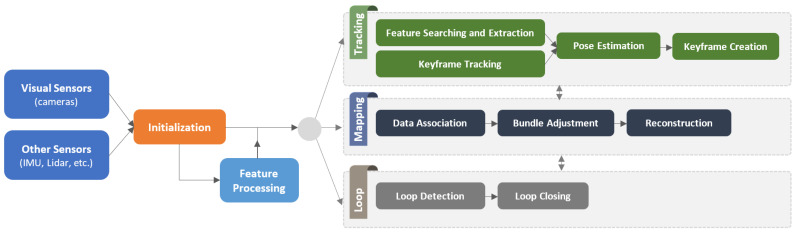
The flowchart of a standard visual SLAM approach. Regarding the direct/indirect methodology utilized, the functionality of some of these modules may change or be ignored. (Re-positioned).

To summarize, Figure 1 shows the overall architecture of a standard VSLAM approach. Accordingly, the system’s inputs may also integrate with other sensor data, such as the Inertial Measurement Unit (IMU) and LiDAR, to provide more information rather than visual data. Moreover, regarding the *direct* or *indirect* methodology used in a VSLAM pipeline, the functionality of the visual feature processing module might be changed or ignored. For instance, the “Feature Processing” stage is only employed in *indirect* approaches to process visual features obtained from the scene. Another factor is utilizing particular modules such as loop closing detection and bundle adjustment for improved execution.

This paper surveys fifty VSLAM works and classifies them into various categories according to diverse aspects. The authors hope their work will present a reference for the robotics community researchers working to improve VSLAM techniques. The rest of the paper is organized as follows: Other published surveys are introduced and discussed in the VSLAM domain in Section 2. Section 3 reviews the evolutionary stages of VSLAM methods that led to the currently existing systems. An abstract level of various VSLAM modules is presented in Section 4, and a classification of the state-of-the-art based on the main contributions is available in Section 5. The authors will then discuss unresolved challenges and potential trends in this field in Section 6. The paper finally concludes in Section 7.

## 2. Related Surveys

There are various survey papers available in the domain of VSLAM that present a general review of the different existing approaches. Each of these papers reviews the major advantages and disadvantages of employing VSLAM approaches. Macario Barros et al. [17] divided approaches into three different classes: visual-only (monocular), visual-inertial (stereo), and RGB-D. They also proposed various criteria for simplifying and analyzing VSLAM algorithms. However, they did not include other vision sensors, such as event camera-based ones, which will be discussed later in Section 4.1. Chen et al. [18] reviewed a wide range of traditional and semantic VSLAM publications. They divided the SLAM development era into *classical*, *algorithmic-analysis*, and *robust-perception* stages and introduced hot issues there. They also summarized classical frameworks that employ direct/indirect methodologies and investigated the impact of deep learning algorithms on semantic segmentation. Although their work provides a comprehensive study of the advanced solutions in this domain, the classification of approaches is only restricted to the *feature types* employed in feature-based VSLAMs. Jia et al. [19] surveyed numerous manuscripts and presented a brief comparison between graph optimization-based methods and deep learning-equipped approaches. Despite presenting a proper comparison among various works, their discussion cannot be generalized due to reviewing a limited number of papers. In another work, Abaspur Kazerouni et al. [20] covered various VSLAM methods, utilized sensory equipment, datasets, and modules and simulated several indirect approaches for comparison and analysis. However, they contribute only to the feature-based algorithms—e.g., *HOG, Scale-Invariant Feature Transform (SIFT), and Speeded Up Robust Features (SURF)*—and deep learning-based solutions and did not cover direct techniques. Bavle et al. [21] analyzed the situational awareness aspects in various SLAM and VSLAM applications and discussed their missing points. They could conclude that operating the lacking situational awareness features could enhance the performance of the current research works.

Other surveys that studied the latest VSLAM approaches focused only on a particular topic or trend. For instance, Duan et al. [15] investigated the progress of deep learning in visual SLAM systems for transportation robotics. The authors summarized the advantages and drawbacks of utilizing various deep learning-based methods in VO and loop closure detection tasks in their paper. The significant advantage of using deep learning approaches in VSLAMs is the accurate feature extraction in pose estimation and the overall performance calculation. In another work in the same field, Arshad and Kim [22] focused on the impact of deep learning algorithms in loop closure detection using visual data. They reviewed various VSLAM papers and analyzed the long-term autonomy of robots in different conditions. Singandhupe and La [23] reviewed the impact of VO and VSLAM in driverless vehicles. They collected approaches that have been evaluated on the KITTI dataset, enabling them to have a brief description of the advantages and demerits of each system. Cheng et al. [18] reviewed the VSLAM-based autonomous driving systems and raised the future development trends of such systems in a similar manuscript. Some other researchers surveyed VSLAM works with the ability to work in real-world conditions. For instance, Saputra et al. [24] targeted the variations of VSLAM techniques operating in dynamic and rough environments and discussed the reconstruction, segmentation, tracking, and parallel execution of threads problems.

Regarding the mentioned surveys, the current survey has particularities that set it apart from other surveys presented so far and provides a comprehensive review of the VSLAM systems presented in different venues. With this, the Research Questions (RQs) that this survey aims to answer can be formulated according to the following dimensions:

- **RQ1:** How can the state-of-the-art methods be classified according to their part in the VSLAM domain and the objectives they try to achieve?
- **RQ2:** What contemporary directions of VSLAM methodologies have the recent publications focused on more?
- **RQ3:** What are the preferred evaluation criteria in recent works regarding the test environment and benchmarks?

The solutions to the first research question will be covered in Section 5, while **RQ2** and **RQ3** will be addressed in Section 6. In this regard, the major contributions of this survey compared to other available VSLAM surveys are:

- Categorizing various recent VSLAM publications regarding the main contributions, criteria, and objectives of researchers in proposing new solutions,
- Analyzing the current trends of VSLAM systems by profoundly investigating different approaches regarding dissimilar aspects,
- And introducing the potential contributions of VSLAM for researchers.

## 3. Evolution of Visual SLAM Algorithms

**Figure 2:**
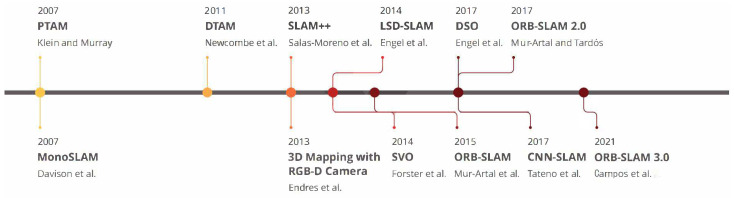
Milestones of highly impactful Visual SLAM approaches. (Re-positioned) [14,25,28,29,30,32,33,34,35,36,37,38].

VSLAM systems have matured over the past years, and several frameworks have played a vital role in this development process. To provide a general picture, Figure 2 illustrates the milestones of the widely referred to as VSLAM approaches that impacted the community and have been used as baselines for other frameworks.

Accordingly, the first endeavor in the literature to implement a real-time monocular VSLAM system was developed by Davison et al. in 2007, where they introduced a framework titled *Mono-SLAM* [25]. Their indirect framework could estimate the camera motion and 3D elements found in the world using the Extended Kalman Filter (EKF) algorithm [26]. *Mono-SLAM* began the primary action in the VSLAM domain despite the lack of global optimization and loop closure detection modules. However, the maps reconstructed by this method only include landmarks and do not offer further detailed information about the area. Klein et al. in [14] proposed Parallel Tracking and Mapping (PTAM) in the same year, in which they divided the entire VSLAM system into two primary threads: *tracking* and *mapping*. This multi-threading baseline was approved by many subsequent works in later works, which will be discussed in this paper. The main idea of their approach was to reduce the computational cost and apply parallel processing to achieve real-time performance. While the *tracking* thread estimates camera motion in real-time, *mapping* predicts the 3D positions of feature points. PTAM was also the first approach to utilize Bundle Adjustment (BA) for jointly optimizing the camera poses and the created 3D map. It uses the Features from Accelerated Segment Test (FAST) [27] corner detector algorithm for keypoints matching and tracking. Despite better performance than *Mono-SLAM*, the algorithm has a complex design and requires user input in the first stage.

A direct approach for measuring depth values and motion parameters for map construction was Dense Tracking and Mapping (DTAM) introduced by Newcombe et al. [28] in 2011. DTAM was a real-time framework equipped with *dense mapping* and *dense tracking* modules and could determine camera poses by aligning the entire frames with a given depth map. To construct the environment map, the mentioned stages estimate the depth of the scene and the motion parameters, respectively. Although DTAM can provide a detailed presentation of the map, it has a high computational cost to perform in real time. As another indirect approach in the domain of 3D mapping and pixel-based optimization, Endres et al. [29], in 2013 proposed a method that could work with RGB-D cameras. Their method performs in real time and is focused on low-cost embedded systems and small robots, but it cannot produce accurate results in featureless or challenging scenarios. In the same year, Salas-Moreno et al. [30] proposed one of the first endeavors of utilizing semantic information in a real-time SLAM framework, titled SLAM++. Their system employs RGB-D sensor outputs and performs 3D camera pose estimation and tracking to shape a pose graph. A pose graph is a graph in which the nodes represent pose estimates and are connected by edges representing the relative poses between nodes with measurement uncertainty [31]. The predicted poses will then be optimized by merging the relative 3D poses obtained from semantic objects in the scene.

With the ripening of the baseline of VSLAM, researchers focused on improving the performance and precision of these systems. In this regard, Forster et al. in 2014, proposed a hybrid VO approach known as Semi-direct Visual Odometry (SVO) [32] as a part of VSLAM architectures. Their method could merge feature-based and direct approaches to perform sensors’ motion estimation and mapping tasks. SVO could work with both monocular and stereo cameras and was equipped with a pose refinement module to minimize re-projection errors. However, the main drawbacks of SVO are employing a short-term data association and the inability to perform loop closure detection and global optimization. LSD-SLAM [33] is another influential VSLAM method introduced by Engel et al. in 2014 and contains *tracking*, *depth map estimation*, and *map optimization* threads. The method could reconstruct large-scale maps using its pose-graph estimation module and was equipped with global optimization and loop closure detection. The weakness of LSD-SLAM is its challenging initialization stage that requires all points in a plane, making it a computationally intensive approach.

Mur-Artal et al. introduced two accurate indirect VSLAM approaches that have attracted the attention of many researchers so far: ORB-SLAM [34] and ORB-SLAM 2.0 [35]. These methods can accomplish localization and mapping in well-textured sequences and perform high-performance position recognition using Oriented FAST and Rotated BRIEF (ORB) features. The first version of ORB-SLAM is able to compute both the camera position and the environment’s structure using the keyframes collected from camera locations. The second version is the extension to ORB-SLAM with three parallel threads, including *tracking* for finding feature correspondences, *local mapping* for map management operations, and *loop closing* for detecting new loops and correcting the drift error. Although ORB-SLAM 2.0 can work with both monocular and stereo camera setups, it cannot be used for autonomous navigation due to reconstructing maps with unknown scales. Another drawback of this approach is its inability to work in texture-less areas or environments with repetitive patterns. The most recent version of this framework, named ORB-SLAM 3.0, was proposed in 2021 [36]. It works with various camera types, such as monocular, RGB-D, and stereo-vision, and provides improved pose estimation outputs.

In recent years and with the significant influences of deep learning in various domains, deep neural network-based approaches could resolve many issues by providing higher recognition and matching rates. Similarly, replacing hand-crafted with learned features in VSLAM is one of the solutions suggested by many recent deep learning-based methods. In this regard, Tateno et al. presented an approach based on Convolutional Neural Networks (CNNs) that processes the input frames for camera pose estimation and uses keyframes for depth prediction, anointed CNN-SLAM [37]. Segmenting camera frames into smaller sections to provide a better understanding of the environment is one of the ideas in CNN-SLAM to provide parallel processing and real-time performance. As a different methodology, Engel et al. also introduced a new trend in direct VSLAM algorithms titled Direct Sparse Odometry (DSO) [38] that merges a direct approach and sparse reconstruction to extract the highest intensity points in image blocks. By tracking sparse sets of pixels, it considers the image formation parameters and uses an indirect tracking method. It should be noted that DSO can only provide perfect accuracy if photometrically calibrated cameras are used and fail to achieve high-accuracy results using regular cameras.

To recap, milestones in the VSLAM systems evolution process reveal that recent approaches focus on the parallel execution of multiple dedicated modules. These modules shaped the general-purpose techniques and frameworks compatible with a broad range of sensors and environments. The mentioned characteristic enables them to be executable in real-time and be more flexible in terms of performance improvement.

## 4. VSLAM Setup

According to the previous section, where the evolution of VSLAM algorithms was studied, adding and improving various modules improved the architecture of the formerly existing techniques and enriched the approaches introduced afterward. Thus, this section investigates multiple configurations that can be found in current VSLAM methodologies. Considering different setups, pipelines, and available configurations, current approaches can be classified into the categories mentioned below:

### 4.1. Sensors and Data Acquisition

The early-stage implementation of a VSLAM algorithm introduced by Davison et al. [25] was equipped with a monocular camera for trajectory recovery. Monocular cameras are the most common vision sensors for a wide range of tasks, such as object detection and tracking [39]. Stereo cameras, on the other hand, contain two or more image sensors, enabling them to perceive depth in the captured images, which leads to more accurate performance in VSLAM applications. These camera setups are cost-efficient and provide informative perception for higher accuracy demands. RGB-D cameras are other variations of visual sensors used in VSLAMs and supply both the depth and colors in the scene. The mentioned vision sensors can provide rich information about the environment in straightforward circumstances—e.g., proper lighting and motion speed—but they often struggle to cope with conditions where the illumination is low or the dynamic range in the scene is high.

In recent years, event cameras have also been used in various VSLAM applications. These low-latency bio-inspired vision sensors generate pixel-level brightness changes instead of standard intensity frames when a motion is detected, leading to a high dynamic range output with no motion blur impact [40]. In contrast with standard cameras, event-based sensors supply trustworthy visual information during high-speed motions and wide-range dynamic scenarios but fail to provide sufficient information when the motion rate is low. Although event cameras can outperform standard visual sensors in severe illumination and dynamic range conditions, they mainly generate unsynchronized information about the environment. This makes traditional vision algorithms unable to process the outputs of these sensors [41]. Additionally, using the spatio-temporal windows of events along with the data obtained from other sensors can provide rich pose estimation and tracking information.

Moreover, some approaches use a multi-camera setup to counter the common issues of working in a real-world environment and improve localization precision. Utilizing multiple visual sensors aid in situations where complicated problems such as occlusion, camouflage, sensor failure, or sparsity of trackable texture occur by providing cameras with overlapping fields of view. Although multi-camera setups can resolve some data acquisition issues, camera-only VSLAMs may face various challenges, such as motion blur when encountering fast-moving objects, features mismatching in low or severe illumination, dynamic object ignorance in scenarios with high pace changes, etc. Hence, some VSLAM applications may be equipped with multiple sensors alongside cameras. Fusing the events and standard frames [42] or integrating other sensors, such as LiDARs [43] and IMUs, to VSLAM are some of the existing solutions.

### 4.2. Visual Features

As discussed in Section 1, detecting visual features and utilizing feature descriptor information for pose estimation is an inevitable stage of indirect VSLAM methodologies. These approaches employ various feature extraction algorithms to understand the environment better and track the feature points in consecutive frames. The feature extraction stage contains a wide range of algorithms, including SIFT [44], SURF [45], FAST [27], Binary Robust Independent Elementary Features (BRIEF) [46], ORB [47], etc. Among them, ORB features have the advantage of fast extraction and matching without losing huge accuracy compared to SIFT and SURF [48].

The problem with some of the mentioned methods is that they cannot effectively adapt to various complex and unforeseen situations. Thus, many researchers have employed CNNs to extract deep-seated features of images for various stages, including VO, pose estimation, and loop closure detection. These techniques may represent supervised or unsupervised frameworks according to the functionality of the methods.

### 4.3. Target Environments

As a strong presumption in many traditional VSLAM practices, the robot works in a static world with no sudden or unanticipated changes. Consequently, although many systems could demonstrate a successful application in specific settings, some unexpected changes in the environment (e.g., the existence of moving objects) are likely to cause complications for the system and degrade the state estimation quality to a large extent. Systems that work in dynamic environments usually employ algorithms such as Optical Flow or Random Sample Consensus (RANSAC) [49] to detect movements in the scene, classify the moving objects as outliers, and skip them while reconstructing the map. Such systems utilize either *geometry/semantic* information or try to improve the localization scheme by combining the results of these two [50].

Additionally, different environments are classified into *indoor* and *outdoor* categories as a general taxonomy. An *outdoor* environment can be an *urban* area with structural landmarks and massive motion changes, such as buildings and road textures, or an *off-road* zone with a weak motion state, such as moving clouds and vegetation, the texture of the sand, etc. As a result of this, the amount of trackable points in *off-road* environments is less than the *urban* areas, which increases the risk of localization and loop closure detection failure. *Indoor* environments, on the other hand, contain scenes with entirely different global spatial properties, such as corridors, walls, and rooms. It should be anticipated that while a VSLAM system might work well in one of the mentioned zones, it might not show the same performance in other environments.

### 4.4. System Evaluation

While some of the VSLAM approaches, especially those with the capability of working in dynamic and challenging environments, are tested on robots in real-world conditions, many research works have used publicly available datasets to demonstrate their applicability. In this regard, the *RAWSEEDS Dataset* by Bonarini et al. [51] is a well-known multi-sensor benchmarking tool containing indoor, outdoor, and mixed robot trajectories with ground-truth data. It is one of the oldest publicly available benchmarking tools for robotic and SLAM purposes. *Scenenet RGB-D* by McCormac et al. [52] is another favored dataset for scene understanding problems, such as semantic segmentation and object detection, containing five million large-scale rendered RGB-D images. The dataset also contains pixel-perfect ground-truth labels and exact camera poses and depth data, making it a potent tool for VSLAM applications. Many recent works in the domain of VSLAM and VO have tested their approaches on the *Technische Universität München (TUM) RGB-D* dataset [53]. The mentioned dataset and benchmarking tool contain color and depth images captured by a Microsoft Kinect sensor and their corresponding ground-truth sensor trajectories. Further, *NTU VIRAL* by Nguyen et al. [54] is a dataset collected by a UAV equipped with a 3D LiDAR, cameras, IMUs, and multiple Ultra-widebands (UWBs). The dataset contains indoor and outdoor instances and is targeted for evaluating autonomous driving and aerial operation performances.

Moreover, the *European Robotics Challenge (EuRoC) Micro Aerial Vehicle (MAV)* dataset by Burri et al. [55] is another popular dataset containing images captured by a stereo camera, along with synchronized IMU measurements and motion ground truth. The collected data in *EuRoC MAV* are classified into easy, medium, and difficult categories according to the surrounding conditions. *OpenLORIS-Scene* by Shi et al. [56] is another publicly available dataset for VSLAM works, containing a wide range of data collected by a wheeled robot equipped with various sensors. It provides proper data for monocular and RGB-D algorithms, along with odometry data from wheel encoders. As a more general-purpose dataset used in VSLAM applications, *KITTI* [57] is a popular collection of data captured by two high-resolution RGB and grayscale video cameras on a moving vehicle. *KITTI* provides accurate ground truth using GPS and laser sensors, making it a highly popular dataset for evaluation in mobile robotics and autonomous driving domains. TartanAir [58] is another benchmarking dataset for the evaluation of SLAM algorithms under challenging scenarios. Additionally, the Imperial College London and National University of Ireland Maynooth (ICL-NUIM) [59] dataset is another VO dataset containing handheld RGB-D camera sequences, considered a benchmark for many SLAM works.

In contrast with the previous datasets, some other datasets contain data acquired using particular cameras instead of regular ones. For instance, the *Event Camera Dataset* introduced by Mueggler et al. [60] is a dataset with samples collected using an event-based camera for high-speed robotic evaluations. Dataset instances contain inertial measurements and intensity images captured by a motion-capture system, making it a suitable benchmark for VSLAMs equipped with event cameras.

**Figure 3:**
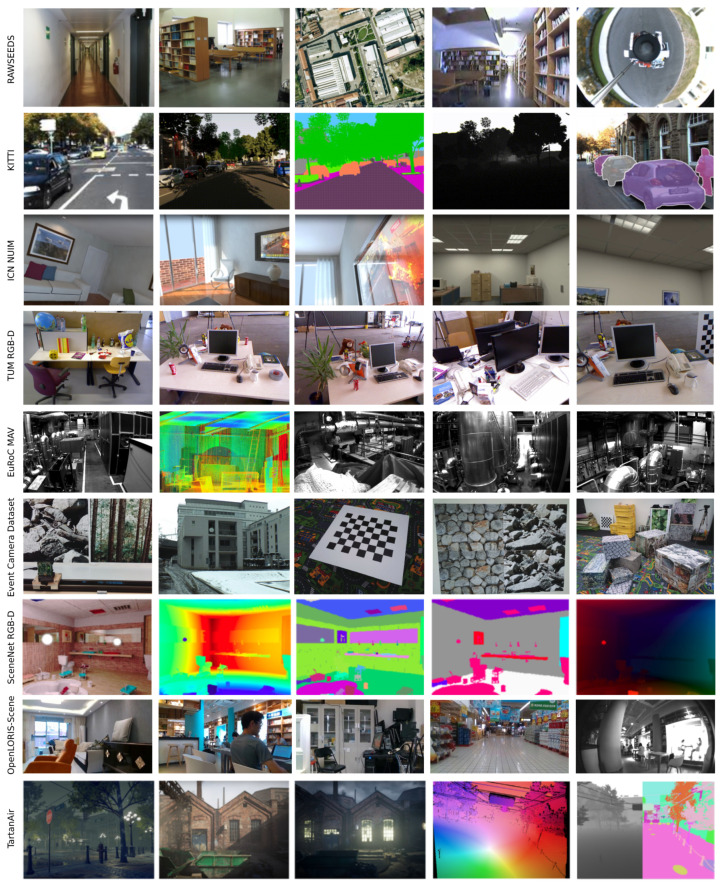
Instances of some of the most popular visual SLAM datasets used for evaluation in various papers. The characteristics of these datasets can be found in Table 1.

The mentioned datasets are used in multiple VSLAM methodologies according to their sensor setups, applications, and target environments. These datasets mainly contain cameras’ extrinsic and intrinsic calibration parameters and ground-truth data. The summarized characteristics of the datasets and some instances of each are shown in Table 1 and Figure 3, respectively.

### 4.5. Semantic Level

Semantic information is required for the robot to understand the scene around it and make more profitable decisions. In many recent VSLAM works, adding semantic-level information to the geometry-based data is preferred to the pure geometry-based approaches, enabling them to deliver conceptual knowledge of the surroundings [61]. In this regard, a pre-trained object recognition module can add semantic information to the VSLAM models [62]. One of the most recent approaches is employing CNNs in VSLAM applications. In general, semantic VSLAM approaches contain four primary components described below [43]:

- *Tracking module:* it uses the two-dimensional feature points extracted from consecutive video frames to estimate the camera pose and construct three-dimensional map points. The calculation of the camera pose and construction of the 3D map points build the baselines of the localization and mapping processes, respectively.
- *Local mapping module*: by processing two sequential video frames, a new 3D map point is created, which is used along with a BA module for an improved camera pose.
- *Loop closing module*: by comparing the keyframes to the extracted visual features and assessing the similarities between them, it tunes the camera pose and optimizes the constructed map.
- *Non-Rigid Context Culling (NRCC)*: the main goal of employing NRCC is to filter temporal objects from video frames in order to reduce their detrimental impact on the localization and mapping stages. It mainly contains a masking/segmentation process for separating various unstable instances in frames, such as people. Since it leads to a lower number of feature points to be processed, NRCC simplifies the computational part and results in a more robust performance.

Accordingly, utilizing the semantic level in VSLAM approaches can improve the uncertainty of pose estimation and map reconstruction. However, the current challenge here is to correctly use the extracted semantic information without hugely impacting the computational cost.

## 5. VSLAM State-of-the-Arts

Various researchers have proposed their approaches and contributions to the VSLAM community in recent years regarding the presented setups studied in the previous section. This section investigates how state-of-the-art techniques can be classified according to their contributions to the VSLAM domain (**RQ1**). In order to pinpoint VSLAM approaches that achieve rich outcomes and present robust architectures, the authors collected and filtered out highly cited publications published in top-notch venues in recent years from Google Scholar (https://scholar.google.com/, accessed on 15 October 2022) and well-known Computer Science bibliography databases: Scopus (https://www.dblp.org/, accessed on 15 October 2022) and DBLP (https://www.scopus.com, accessed on 15 October 2022). The manuscripts referred to in the mentioned publications studied and the ones most relevant to the VSLAM domain are purified. After exploring the papers, the collected publications are categorized based on their main objectives to solve particular problems into sub-sections presented below:

### 5.1. Objective I: Multi-Sensor Processing

This category covers the range of VSLAM approaches that employ several sensors instead of a single camera to acquire data and understand the environment better. In this regard, while some techniques rely on *only cameras* as the operated visual sensors, others combine *various sensors* to enhance the accuracy of their algorithms.

#### 5.1.1. Multiple Cameras

As it might be difficult to recreate the 3D trajectories of moving objects with a single camera, some researchers suggest using multiple cameras instead. For instance, *CoSLAM* (https://github.com/danping/CoSLAM, accessed on 15 October 2022) is a VSLAM system introduced by Zou and Tan [63] uses separate cameras deployed on various platforms to reconstruct robust maps. Their system combines multiple cameras moving around independently in a dynamic environment and reconstructs the map regarding their overlapping fields of view. The process makes it easier to rebuild dynamic points in 3D by mixing intra- and inter-camera pose estimation and mapping. *CoSLAM* tracks visual features using the Kanade-Lucas-Tomasi (KLT) algorithm and operates in static and dynamic contexts, including indoors and outdoors, where the relative positions and orientations may shift over time. The primary drawback of this method is it requires sophisticated hardware to interpret numerous camera outputs and increases computational cost by adding more cameras.

For challenging off-road settings, Yang et al. [64] developed a multi-camera cooperative panoramic vision VSLAM approach. Their approach gives each camera independence to increase the performance of the VSLAM system under challenging conditions, such as occlusion and texture sparsity. In order to determine the matching range, they extract ORB features from cameras’ overlapping fields of view. Additionally, they employed a deep learning technique based on a CNN to recognize similar features for loop closure detection. For the experiment, the authors used a dataset produced by a panoramic camera and an integrated navigation system.

*MultiCol-SLAM* is another open-source VSLAM framework with multi-camera configurations by Urban and Hinz [65]. They use their previously created model, *MultiCol*, to enhance ORB-SLAM utilizing a keyframe-based process that supports multiple fisheye cameras. They added a Multi-Keyframes (MKFs) processing module to ORB-SLAM, which collects and turns images into keyframes. The authors also proposed the idea of multi-camera loop closing, in which loop closures are detected from MKFs. Although their method operates in real-time, it requires significant computer power because several threads must run simultaneously.

#### 5.1.2. Multi-Modal Sensors

Other approaches proposed fusing various sensor modalities and combining vision- and inertial-based sensor outputs for better performance. In this regard, a low-cost, indirect LiDAR-assisted VSLAM called *CamVox* (https://github.com/ISEE-Technology/CamVox, accessed on 15 October 2022) was proposed by Zhu et al. [66] and demonstrated reliable performance and accuracy. Their method uses ORB-SLAM 2.0 and combines the unique capabilities offered by Livox LiDARs as the premium depth sensors with the outputs from RGB-D cameras. The authors used an IMU to synchronize and correct the non-repeating scanned locations. Their contribution is presenting an autonomous LiDAR-camera calibration method that operates in uncontrolled environments. Real-world tests on a robot platform indicate that *CamVox* performs in real time while processing the environment.

The authors of [67] proposed a multi-modal system titled *VIRAL (Visual-Inertial-Ranging-LiDAR) SLAM* that couples camera, LiDAR, IMU, and UWB. They also presented a map-matching marginalization scheme for visual features based on the local map constructed from LiDAR point clouds. The visual components are extracted and tracked using a BRIEF algorithm. The framework also contains a synchronization scheme and trigger for the utilized sensors. They tested their approach on simulation environments, and their generated dataset, titled NTU VIRAL [54], which contains data captured by camera, LiDAR, IMU, and UWB sensors. However, their approach is computationally intensive due to handling synchronization, multi-threading, and sensor conflict resolution.

Vidal et al. [42] proposed integrating events, camera frames, and IMU in parallel configurations for reliable position estimation in high-speed settings. Their *Ultimate SLAM* (https://github.com/uzh-rpg/rpg_ultimate_slam_open, accessed on 15 October 2022) system is based on an event camera and a keyframe-based nonlinear optimization pipeline introduced in [68]. They use the FAST corner detector and the Lucas–Kanade tracking algorithm for feature detection and tracking, respectively. *Ultimate SLAM* avoids motion blur problems brought on by high-speed activity and operates in dynamic situations with varied lighting conditions. The efficiency of this technique on the “Event Camera Dataset” was obvious in comparison to alternative event-only and conventional camera configurations. The authors also tested Ultimate SLAM on an autonomous quadrotor equipped with an event camera to show how their system can manage flight conditions that are impossible for conventional VO platforms to handle. The major challenge in *Ultimate SLAM* is the synchronization of events with standard frame outputs.

A tightly-coupled monocular camera and UWB range sensors were suggested by Nguyen et al. [69] for VSLAM. They use a combination of feature-based (visible) and feature-less (UWB) landmarks to create a map. It operates effectively when UWB is exposed to multi-path effects in congested surroundings. They built their indirect method on ORB-SLAM and employed ORB features for pose estimation. They tested their system on a generated dataset with hand-carried movements simulating an employed aerial robot. The synchronization of the camera and UWB sensor is one of the difficulties in this case, but it has been overcome by employing a new camera pose with its related timestamp for each new image.

### 5.2. Objective II: Pose Estimation

Methods classified in this category focus on improving the pose estimation of a VSLAM approach using various algorithms. The primary patterns observed in this section contain utilizing points and lines noticeable in the scene, employing particular features such as fiducial markers’ data, and using deep learning to extract correct poses.

#### 5.2.1. Lines/Points Data

In this regard, Zhou et al. [70] suggested employing building structural lines as useful features to determine the camera pose. Structural lines are associated with dominant directions and encode global orientation information, resulting in improved predicted trajectories. *StructSLAM*, the mentioned method, is a 6-Degree of Freedom (DoF) VSLAM technique that operates in both low-feature and featureless conditions. It employs EKF to estimate variables based on the current directions in the scene. For evaluation, the indoor scenes dataset from RAWSEEDS 2009 and a set of generated sequential image datasets were used.

*Point and Line SLAM (PL-SLAM)* (https://github.com/HarborC/PL-SLAM, accessed on 15 October 2022), a VSLAM system based on ORB-SLAM optimized for non-dynamic low-texture settings, was introduced by Pumarola et al. [71]. The system simultaneously fuses line and point features for improved posture estimation and helps running in situations with few feature points. The authors tested *PL-SLAM* on their generated dataset and TUM RGB-D. The drawback of their method is the computational cost and the essence of using other geometric primitives, e.g., planes, for a more robust accuracy.

Gomez-Ojeda et al. [72] introduced *PL-SLAM* (https://github.com/rubengooj/pl-slam, accessed on 15 October 2022) (different from the framework with the same name by Pumarola et al. in [71]), an indirect VSLAM technique that uses points and lines in stereo vision cameras to reconstruct an unseen map. They merged segments obtained from points and lines in all VSLAM modules with visual information taken from successive frames in their approach. Using the ORB and Line Segment Detector (LSD) algorithms, points and line segments are retrieved and tracked in subsequent stereo frames in *PL-SLAM*. The authors tested *PL-SLAM* on EuRoC and KITTI datasets and could outperform the stereo version of ORB-SLAM 2.0 in terms of performance. One of the main drawbacks of *PL-SLAM* is the computational time required for the feature tracking module and considering all structural lines to extract information about the environment.

A degeneracy avoidance technique for monocular point- and line-based VSLAM systems was introduced by Lim et al. [73]. Another contribution of their work is introducing an Optical Flow-based line tracking module that extracts line characteristics, filters out the short lines in each frame, and matches the previously identified ones. To demonstrate the efficacy of their technique compared to the established point-based approaches, they tested their system on the EuRoC MAV dataset. Their system lacks an adaptive approach to identify the correct optimization parameters, notwithstanding the strong findings.

#### 5.2.2. Advanced Features

*Dual Quaternion Visual SLAM (DQV-SLAM)*, a framework for stereo-vision cameras that uses a broad Bayesian framework for 6-DoF posture estimation, was proposed in [74]. In order to prevent the linearization of the nonlinear spatial transformation group, their approach uses progressive Bayes updates. For point clouds of maps and Optical Flow, *DQV-SLAM* uses ORB features to enable reliable data association in dynamic circumstances. On the KITTI and EuRoC datasets, the method could estimate experiment results reliably. However, it lacks a probabilistic interpretation for the stochastic modeling of poses and is computationally demanding for sampling approximation-based filtering.

Muñoz-Salinas et al. [75] developed a technique using artificial squared planar markers to recreate a large-scale interior environment map. Their real-time *SPM-SLAM* system can solve the ambiguity issue of pose estimation using markers if at least two of them are visible in each video frame. They created a dataset with video sequences of markers placed in two rooms joined by a door for examination. Although *SPM-SLAM* is cost-effective, it only works when numerous planar markers are scattered around the area while at least two are visible for marker connection recognition. Moreover, the ability of their framework to handle dynamic changes in the scene is not measured.

#### 5.2.3. Deep Learning

In another approach, Bruno and Colombini [76] proposed *LIFT-SLAM*, which combines deep learning-based feature descriptors with conventional geometry-based systems. They expanded the ORB-SLAM system’s pipeline and employed a CNN to extract features from images, using the learned features to provide more dense and precise matches. For purposes of detection, description, and orientation estimation, *LIFT-SLAM* fine-tunes a Learned Invariant Feature Transform (LIFT) deep neural network. Studies using the KITTI and EuRoC MAV datasets’ indoor and outdoor instances revealed that *LIFT-SLAM* outperforms conventional feature-based and deep learning-based VSLAM systems in terms of accuracy. However, the weaknesses of the method are its computationally intensive pipeline and un-optimized CNN design, which leads to near real-time performance.

Naveed et al. [77] proposed another deep learning-based VSLAM solution with a reliable and consistent module, even on routes with extreme turns. Their approach outperformed several VSLAMss using a deep reinforcement learning network trained on realistic simulators. Furthermore, they provided a baseline for active VSLAM evaluation and could properly generalize across actual indoor and outdoor environments. The network’s path planner developed the ideal path data, which are received by its base system, ORB-SLAM. The authors also released a dataset with actual navigation episodes in challenging and texture-less environments for evaluation.

As another approach, *RWT-SLAM* is a deep feature matching-based VSLAM framework the authors in [78] proposed for weakly textured situations. Their method, which is based on ORB-SLAM, is fed with feature masks from an enhanced LoFTR [79] algorithm for local image feature matching. A CNN architecture and the LoFTR algorithm were used to extract coarse-level and fine-level descriptors in the scene, respectively. *RWT-SLAM* is examined on the TUM RGB-D and OpenLORIS-Scene datasets, as well as a real-world dataset gathered by the authors. However, their system is computationally demanding despite the robust feature-matching results and performance.

Sun et al. [80] proposed a VSLAM method that creates an objected-oriented semantic point cloud map using integrated tracking and scene segmentation. Their deep learning-based system optimizes camera poses using object constraints and reconstructs multiple objects in 3D space. It is built upon ORB-SLAM 2.0 and utilizes Mask Region-based CNN (Mask R-CNN) [81] to extract 2D semantic information from the scene. Additionally, it uses the Deepsort [82] algorithm for multi-object tracking detected by the semantic feature extractor. Experimental results on the TUM RGB-D dataset after both self-training and using Microsoft COCO [83] weights showed deficient positioning errors. The authors compared their work with other semantic VSLAM methods, claiming their framework can extract semantic information more accurately than previous efforts. However, their system does not work in real-time due to demanding a pre-processing step for segmentation and the slow performance of Mask R-CNN.

### 5.3. Objective III: Real-World Viability

Approaches in this category have the primary objective of being used in various environments and working under several scenarios. The authors noticed that the references in this section are highly integrated with *semantic* information extracted from the environment and present an end-to-end VSLAM application.

#### 5.3.1. Dynamic Environments

A VSLAM system titled *DS-SLAM* (https://github.com/ivipsourcecode/DS-SLAM, accessed on 15 October 2022), introduced by Yu et al. [61], can be used in dynamic contexts and offers semantic-level information for map construction. The system is built upon ORB-SLAM 2.0 and contains five threads: *tracking*, *semantic segmentation*, *local mapping*, *loop closing*, and *dense semantic map construction*. To exclude dynamic items before the pose estimation process and increase localization accuracy, *DS-SLAM* employs the Optical Flow algorithm with a real-time semantic segmentation network called *SegNet* [84]. *DS-SLAM* has been tested in real-world settings and with RGB-D cameras, as well as on the TUM RGB-D dataset. However, despite its high accuracy in localization, it faces semantic segmentation limitations and computationally intensive features.

*Semantic Optical Flow SLAM (SOF-SLAM)*, an indirect VSLAM system built upon the RGB-D mode of ORB-SLAM 2.0, is another method in highly dynamic environments proposed by Cui and Ma [50]. Their approach uses the Semantic Optical Flow dynamic feature detection module, which extracts and skips the changing features concealed in the semantic and geometric information provided by ORB feature extraction. In order to deliver accurate camera pose and environment reports, *SOF-SLAM* makes use of SegNet’s pixel-wise semantic segmentation module. In extremely dynamic situations, experimental findings on the TUM RGB-D dataset and in real-world settings demonstrated that SOF-SLAM performs better than ORB-SLAM 2.0. However, the ineffective method of non-static feature recognition and reliance on just two consecutive frames for this purpose are *SOF-SLAM*’s weakest points.

Using the Optical Flow method to separate and eliminate dynamic feature points, Cheng et al. [85] suggested a VSLAM system for dynamic environments. They have utilized the ORB-SLAM pipeline’s structure and supplied it with fixed feature points generated from typical monocular camera outputs for precise posture estimation. The system indicated operates in featureless circumstances by sorting Optical Flow values and using them for feature recognition. According to experimental results on the TUM RGB-D dataset, the suggested system functions well in dynamic indoor circumstances. However, the system’s configuration uses an offline threshold for motion analysis, making it difficult to use in a variety of dynamic environment situations.

Another VSLAM strategy was released by Yang et al. [86] that reconstructs the environment map using semantic segmentation network data, a motion consistency detection technique, and geometric restrictions. Their approach, which is based on ORB-SLAM 2.0’s RGB-D setting, performs well in dynamic and indoor environments. Only the stable features from the scene are retained using an improved ORB feature extraction technique, while the dynamic characteristics are disregarded. The features and the semantic data will then be combined to create a static semantic map. Evaluation findings on the Oxford and TUM RGB-D datasets demonstrated the effectiveness of their approach in enhancing location accuracy and creating semantic maps with a wealth of data. However, their system can run into problems in corridors or places with less information.

#### 5.3.2. Deep Learning

In another work by Li et al. [87] called *DXSLAM* (https://github.com/ivipsourcecode/dxslam, accessed on 15 October 2022), deep learning is used to find keypoints that resemble SuperPoints and to produce both the general descriptors and the images’ keypoints. They trained a cutting-edge deep CNN titled HF-NET to produce frame- and keypoint-based descriptions by extracting local and global information from each frame. They also used the offline Bag of Words (BoW) method to train a visual vocabulary of local characteristics for precise loop closure recognition. *DXSLAM* operates in real-time without using a Graphics Processing Unit (GPU) and is compatible with contemporary CPUs. Even if such qualities are not specifically addressed, it has a great ability to resist dynamic changes in dynamic contexts. *DXSLAM* has been tested on TUM RGB-D and OpenLORIS-Scene datasets and both indoor and outdoor images and could achieve more accurate results than ORB-SLAM 2.0 and DS-SLAM. However, the major disadvantages of this method are the complex architecture for feature extraction and the incorporation of deep features into an old SLAM framework.

In another approach, Li et al. [88] developed a real-time VSLAM technique for extracting feature points based on deep learning in complicated situations. The method can run on a GPU and supports the creation of 3D dense maps, and is a multi-task CNN for feature extraction with self-supervision capabilities. The CNN output is binary code strings with a fix-length of 256, making it possible to be replaced by more conventional feature point detectors such as ORB. It comprises three threads for reliable and timely performance in dynamic scenarios: *tracking*, *local mapping*, and *loop closing*. The system supports monocular and RGB-D cameras using ORB-SLAM 2.0 as a baseline. The authors tested their methodology on the TUM dataset and two datasets collected in a corridor and an office using a Kinect camera for the experiments.

Steenbeek and Nex [89] present a real-time VSLAM technique that uses a ResNet-50 [90] CNN for accurate scene interpretation and map reconstruction. Their solution utilizes monocular camera streams from a UAV during flight and employs a depth-estimating neural network for reliable performance. The mentioned method is based on ORB-SLAM 2.0 and makes use of visual cues collected from indoor environments. Additionally, the CNN is trained on more than 48,000 indoor examples and operates the pose, space depth, and RGB inputs to estimate scale and depth. The TUM RGB-D dataset and a real-world test using a drone were used to evaluate the system, which demonstrated enhanced pose estimation accuracy. However, the system struggles in situations without texture and needs both CPU and GPU resources for real-time performance.

Su et al. [91] proposed a fast deep learning-based VSLAM system for dynamic environments. Their real-time framework integrates ORB-SLAM 2.0 with the lightweight object detection network YOLOv5s [92], enabling it to acquire semantic information in the scene. Another contribution of their work is introducing an optimized homography matrix module to create more accurate Optical Flow vectors. According to the experiments, their method performs better than the existing semantic VSLAM algorithms in terms of accuracy and performance. They employed the TUM RGB-D dataset to analyze their system’s runtime and accuracy compared to other works. Nevertheless, according to the authors’ claims, the backbone network of YOLOv5 in their approach needs to be optimized yet to make it more robust and practicable.

In another work, with a focus on resolving the challenges of dynamic scenes, Chen et al. [93] proposed a deep learning-based VSLAM approach. They presented a high-precision contour extractor and a lightweight contour optimization algorithm that improves instance segmentation accuracy at a low cost. Their method has been tested on the TUM RGB-D dataset, resulting in robust pose estimation and map construction compared to other VSLAM approaches, such as ORB-SLAM 2.0. Despite the mentioned advantages, whether the proposed approach works fine under complex circumstances is not verified.

#### 5.3.3. Artificial Landmarks

A technique called *UcoSLAM* (https://sourceforge.net/projects/ucoslam/, accessed on 15 October 2022) [94] by Muñoz-Salinas and Medina-Carnicer outperforms conventional VSLAM systems by combining natural and human-made landmarks and automatically calculating the scale of the surroundings using fiducial markers. *UcoSLAMs* primary driving force is to combat natural landmarks’ instability, repetition, and poor tracking qualities. It can operate in surroundings without tags or features since it can operate in keypoints-only, markers-only, and mixed modes. *UcoSLAM*’s tracking mode locates map correspondences, optimizes re-projection errors, and re-localizes in the event of tracking failure. Additionally, it has a marker-based loop closure detection system and can describe features using any descriptor, including ORB and FAST. Despite all the plus points of *UcoSLAM*, the system executes in multiple threads, making it a time-consuming approach for particular scenarios.

#### 5.3.4. Multi-Modal Setups

Another VSLAM strategy for dynamic indoor and outdoor situations is *DMS-SLAM* [95], which supports monocular, stereo, and RGB-D visual sensors. The system employs sliding window and Grid-based Motion Statistics (GMS) [96] feature-matching methods to find static feature locations. Using the ORB-SLAM 2.0 system as its foundation, *DMS-SLAM* tracks the static features recognized by the ORB algorithm. The authors tested their suggested methodology on the TUM RGB-D and KITTI datasets and outperformed cutting-edge VSLAM algorithms. Additionally, because the feature points on the dynamic objects were removed during the tracking step, *DMS-SLAM* performs more quickly than the original ORB-SLAM 2.0. Despite the described positive points, the suggested solution encounters difficulties in situations with little texture, fast motion, and highly dynamic environments.

### 5.4. Objective IV: Resource Constraint

In another category, some of the VSLAM methodologies are built for devices with limited computational resources compared to other standard devices. Approaches in this category either attempt to deliver a lightweight setup enabling them to work in scenarios with constrained hardware or use complementary resources for data offloading. For instance, VSLAM systems designed for mobile devices and robots with embedded systems are included in this category.

#### 5.4.1. Limited Processing Capabilities

*edgeSLAM* is a real-time, edge-assisted semantic VSLAM system for mobile and resource-constrained devices proposed by Xu et al. [97]. It employs a series of fine-grained modules to be used by an edge server and the associated mobile devices rather than requiring heavy threads. A semantic segmentation module based on Mask R-CNN is also included in *edgeSLAM* to improve segmentation and object tracking. The authors put their strategy into practice on an edge server with several commercial mobile devices, such as cell phones and development boards. By reusing the findings of the object segmentation, they avoided duplicate processing by adapting system parameters to different network bandwidth and latency situations. *EdgeSLAM* has been evaluated on monocular vision instances of TUM RGB-D, KITTI, and the created dataset for experimental settings.

For stereo camera setups, Schlegel, Colosi, and Grisetti [98] suggested a lightweight feature-based VSLAM framework titled *ProSLAM* (https://gitlab.com/srrg-software/srrg_proslam, accessed on 15 October 2022) that achieves results on par with cutting-edge techniques. Four modules make up their approach: the *triangulation* module, which creates 3D points and associated feature descriptors; the *incremental motion estimation* module, which processes two frames to determine the current position; the *map management* module, which creates local maps; and the *re-localization* module, which updates the world map based on the similarities of local maps. *ProSLAM* retrieves the 3D position of the points using a single thread and leverages a small number of well-known libraries for a system that is simple to create. According to the experiments on KITTI and EuRoC datasets, their approach can achieve robust results. However, it shows weakness in rotation estimation and does not contain any bundle adjustment modules.

Bavle et al. [99] proposed *VPS-SLAM* (https://github.com/hridaybavle/semantic_slam, accessed on 15 October 2022), a lightweight graph-based VSLAM framework for aerial robotics. Their real-time system integrates geometrical data, several object detection techniques, and visual/visual-inertial odometry for pose estimation and building the semantic map of the environment. Low-level characteristics, IMU measurements, and high-level planar information are all used by VPS-SLAM to reconstruct sparse semantic maps and predict robot states. The system leverages the lightweight version of You Only Look Once v2.0 (YOLO2) [100] trained on the COCO dataset [83] for object detection due to its real-time and computationally effective performance. They used a handheld camera setup and an aerial robotic platform equipped with an RGB-D camera for testing. The TUM RGB-D dataset’s indoor instances were used to test their methodology, and they were able to provide results that were on par with those of well-known VSLAM methods. However, only a small number of objects (e.g., chairs, books, and laptops) can be used by their VSLAM system to build a semantic map of the surrounding area.

Another real-time indoor VSLAM method was proposed by Tseng et al. [101] that requires a low-cost setup. The authors also presented a technique for estimating the number of frames and visual elements required for a reasonable degree of localization accuracy. Their solution is based on the OpenVSLAM [102] framework and makes use of it for emergencies that arise in the real world, such as gaining access to specific targets. The system acquires the scene’s feature map for precise pose estimation by applying the Efficient Perspective-n-Point (EPnP) and RANSAC algorithms. According to tests conducted in a building, their device can deliver accurate findings under difficult lighting conditions.

#### 5.4.2. Computation Offloading

Ben Ali et al. [103] suggested using edge computing to enable the offloading of resource-intensive operations to the cloud and reduce the computational burden on the robot. They modified the architecture of ORB-SLAM 2.0 in their indirect framework, Edge-SLAM (https://github.com/droneslab/edgeslam, accessed on 15 October 2022), by maintaining the tracking module on the robot and delegating the remainder to the edge. By splitting the VSLAM pipeline between the robot and the edge device, the system can maintain both a local and a global map. With fewer resources available, they could still execute properly without sacrificing accuracy. They used the TUM RGB-D dataset and two different mobile devices to generate a custom indoor environment dataset using RGB-D cameras for evaluation. However, one of their approach’s drawbacks is the architecture’s complexity due to the decoupling of various SLAM modules. Another setback is that their system works only in short-term settings, and utilizing Edge-SLAM in long-term scenarios (e.g., multiple days) would face performance degradation.

### 5.5. Objective V: Versatility

VSLAM works categorized in this class are focused on straightforward development, utilization, adaptation, and extension. In this regard, the practices encountered in such VSLAM frameworks is to simplify the development of functional changes for further improvements.

In this regard, Sumikura et al. [102] introduced *OpenVSLAM* (https://github.com/xdspacelab/openvslam, accessed on 15 October 2022), a highly adaptable open-source VSLAM framework that seeks to be quickly developed upon and called by other third-party programs. Their feature-based approach is compatible with multiple camera types, including monocular, stereo, and RGB-D, and can store or reuse the reconstructed maps for later usage. *OpenVSLAM* performs better in terms of tracking accuracy and efficiency than ORB-SLAM and ORB-SLAM 2.0 due to its powerful ORB feature extractor module. However, the open-source code of the system has been discontinued owing to worries over code similarities that infringed on the rights to ORB-SLAM 2.0.

To bridge the gap between real-time capabilities, accuracy, and resilience, Ferrera et al. [104] developed *OV${}^{2}$SLAM* (https://github.com/ov2slam/ov2slam, accessed on 15 October 2022), which works with monocular and stereo-vision cameras. By limiting the extraction of features to keyframes and monitoring them in subsequent frames by eliminating photo-metric errors, their method lessens the computational load. In this sense, *OV${}^{2}$SLAM* is a hybrid strategy that combines the virtues of the direct and indirect categories of VSLAM algorithms. Using well-known benchmarking datasets, including EuRoC, KITTI, and TartanAir in both indoor and outdoor experiments, it was demonstrated that *OV${}^{2}$SLAM* surpasses several popular techniques in terms of performance and accuracy.

Another approach in this category, titled *DROID-SLAM* (https://github.com/princeton-vl/DROID-SLAM, accessed on 15 October 2022), a deep learning-based visual SLAM for monocular, stereo, and RGB-D cameras, is proposed by Teed and Deng [105]. They could attain greater accuracy and robustness than well-known monocular and stereo-track methods. Their solution operates in real-time and consists of *back-end* (for bundle adjustment) and *front-end* (for keyframe collection and graph optimization) threads. *DROID-SLAM* has already been taught using monocular camera examples. Therefore, it does not need to be trained again to use stereo and RGB-D inputs. The approach minimizes the projection error, like indirect methods, while not requiring any pre-processing for feature identification and matching. A feature extraction network comprising downsampling layers and residual blocks processes each input image to create dense features. *DROID-SLAM* has been tested on well-known datasets, including TartanAir, EuRoC, and TUM RGB-D, and could achieve acceptable results.

Bonetto et al. in [106] propose *iRotate* (https://github.com/eliabntt/irotate_active_slam, accessed on 15 October 2022), an active technique for omnidirectional robots with RGB-D cameras. Additionally, a module for spotting obstructions in the camera’s area of vision is employed in their approach. By offering observation coverage of previously unexplored places and previously visited locations, *iRotate*’s primary objective is to lessen the distance the robot must go to map the environment. The mentioned method uses a VSLAM framework with graph features as its back-end. The authors could attain outcomes that were on par with those of cutting-edge VSLAM methods by providing comparisons in simulation and on a real three-wheel omnidirectional robot. However, the major weakness of their method is that the robot might face start-stop cases in which the local paths are re-planned.

### 5.6. Objective VI: Visual Odometry

Approaches in this category aim to determine the position and orientation of the robot with the highest possible accuracy. With this, the leading solutions are using deep neural networks to extract visual patterns, process adjacent frames’ data for enhanced tracking, and utilize other feature processing methodologies.

#### 5.6.1. Deep Learning

The *Dynamic-SLAM* framework was proposed in [107], which leverages deep learning for accurate pose prediction and suitable environment comprehension. As part of a semantic-level module for optimized VO, the authors employed a CNN to identify moving objects in the environment, which helped them lower the pose estimate error brought on by improper feature matching. Additionally, *Dynamic-SLAM* uses a selective tracking module to ignore dynamic locations in the scene and a missed feature corrective algorithm for speed invariance in adjacent frames. Despite the excellent results, the system requires huge computational costs and faces the risk of misclassifying dynamic/static objects due to a limited number of defined semantic classes.

Bloesch et al. [108] proposed the *Code-SLAM* (https://github.com/silviutroscot/CodeSLAM, accessed on 15 October 2022) direct technique, which offers a condensed and dense representation of the scene geometry. Their VSLAM system, which only functions with monocular cameras, is an enhanced version of *PTAM* [14]. They divided intensity images into convolutional features and fed them to a depth auto-encoder using a CNN trained on intensity images from the SceneNet RGB-D dataset. Indoor examples of the EuRoC datasets have been used to test *Code-SLAM*, and the findings were promising in terms of accuracy and performance.

*DeepVO* (http://senwang.gitlab.io/DeepVO/, accessed on 15 October 2022), an end-to-end VO framework using a Deep Recurrent Convolutional Neural Network (RCNN) architecture for monocular settings, was proposed by Wang et al. [109]. Their approach uses deep learning to automatically learn the appropriate features, model sequential dynamics and relations, and infer poses directly from color frames. The *DeepVO* architecture includes a CNN called FlowNet for computing Optical Flow from sequential frames and two Long Short-Term Memory (LSTM) layers for estimating the temporal changes based on the feed provided by the CNN. The framework can simultaneously extract visual characteristics and perform sequential modeling by combining CNN and Recurrent Neural Network (RNN). *DeepVO* can incorporate geometry with the knowledge models learned for an enhanced VO. However, it cannot be utilized to replace conventional geometry-based VO approaches.

Parisotto et al. [110] proposed a DeepVO-like end-to-end system using a Neural Graph Optimization (NGO) step instead of LSTMs. Their approach operates a loop closure detection and correction mechanism based on temporally distinct poses. NGO uses two attention-optimization methods to jointly optimize the aggregated predictions made by convolutional layers of local pose estimation modules and delivers global pose estimations. They experimented with their technique on 2D and 3D mazes and outperformed DeepVO’s performance and accuracy levels. The mentioned approach needs to be connected to a SLAM framework to supply the re-localization signals.

In another work, one of the most extensive VSLAM frameworks titled *DeepFactors* (https://github.com/jczarnowski/DeepFactors, accessed on 15 October 2022) was introduced by Czarnowski et al. [111] for densely rebuilding the environment map with monocular cameras. For a more reliable map reconstruction, their real-time solution performs joint optimization of the pose and depth, makes use of probabilistic data and combines learned and model-based approaches. The authors modified the *CodeSLAM* framework and added missing components, such as local/global loop detection. The system is evaluated on the ICL-NUIM and TUM RGB-D datasets after being trained on roughly 1.4 million ScanNet [112] images. *DeepFactors* improves the idea of the CodeSLAM framework and focuses on code optimization in traditional SLAM pipelines. However, due to the computational costs of the modules, this approach requires employing GPUs to guarantee real-time performance.

The authors of [113] proposed a VSLAM approach based on ORB-SLAM that operates well in low-texture and illumination-changing environments pertaining to its deep CNN algorithm. Their system utilizes a Visual Geometry Group (VGG) [114] network for accurate feature point extraction and an improved bundle adjustment optimization that operates pleasingly with VGG-based VO. A set of modifications were considered on the standard VGG architecture to keep the approach real-time. They trained the network on an indoor image dataset collected by a wheeled robot equipped with a monocular camera and an IMU. The experiments showed that the proposed approach was very good when examining feature detection and matching functionalities in various illumination conditions. It should be noted that despite the accurate feature matching, the experiments needed to be verified on public datasets and outdoor settings.

Gu et al. [115] proposed a modified version of ORB-SLAM that uses deep binary local descriptors provided by a CNN instead of ORB features. Their approach, *DBLD-SLAM*, can improve positioning and VO accuracy by utilizing higher-quality feature matching and tracking, leading to enhanced adaptive scale, quantization, and correlation losses. Experiments on the Tartanair and HPatches [116] datasets showed that *DBLD-SLAM* achieves better results than other VSLAM methodologies with hand-craft feature processing. Despite the real-time performance of the proposed system, some revisions are required to decrease its computational cost.

#### 5.6.2. In-Depth Adjacent Frame Processing

By reducing the photometric and geometric errors between two pictures for camera motion detection, the authors of [117] (https://vision.in.tum.de/data/software/dvo, accessed on 15 October 2022) developed a real-time dense SLAM approach for RGB-D cameras, improving their prior method [118]. Their keyframe-based solution expands *Pose SLAM* [119] that only keeps non-redundant poses for producing a compact map, adds dense visual odometry characteristics, and effectively utilizes the information from camera frames for a reliable camera motion estimation. The authors also employed an entropy-based technique to gauge keyframe similarity for loop closure detection and drift avoidance. However, their approach still needs work in the areas of loop closure detection and keyframe selection quality.

In another work introduced by Li et al. [120], real-time dynamic object removal is accomplished using a feature-based VSLAM approach known as *DP-SLAM*. This method uses a Bayesian probability propagation model based on the likelihood of the keypoints derived from moving objects. The variation of geometry restrictions and semantic data can be overcome by *DP-SLAM* using the moving probability propagation algorithm and iterative probability updates. It is integrated with ORB-SLAM 2.0 and has been tested on the TUM RGB-D dataset. Despite the accurate results, the system only works in sparse VSLAMs and faces high computational costs due to the iterative probability updater module.

*Pair-Navi*, a suggested indoor navigation system by Dong et al. [121], reuses a previously traced path by an agent for usage in the future by other agents. Hence, a previous traveler, known as the *leader*, captures the trace information, such as turnings and particular ambient qualities, and gives it to a later *follower* that needs to travel to the same destination. While the *follower* uses a re-localization module to determine its location concerning the reference trace, the *leader* incorporates visual odometry and trajectory creation modules. The system employs Mask R-CNN to recognize and remove dynamic objects from the video feature set. They tested *Pair-Navi* on a set of generated datasets and several smartphones for the experiments.

#### 5.6.3. Various Feature Processing

A text-based VSLAM system called *TextSLAM* proposed by Li et al. [122] incorporates text items retrieved from the scene using the FAST corner detection technique in the SLAM pipeline. Texts include a variety of textures, patterns, and semantic meanings, making the approach more efficient to use them to create 3D text maps of high quality. *TextSLAM* uses texts as reliable visual fiducial markers, parametrizes them after the first frame in which they are found, and then projects the 3D text object onto the target image to locate it again. They also presented a new three-variable parameterization technique for initializing instantaneous text features. Using a monocular camera and a dataset created by the authors, experiments were conducted in indoor and outdoor settings. Operating in text-free surroundings, interpreting short letters, and requiring the storage of enormous text dictionaries are the three fundamental challenges of *TextSLAM*.

Xu et al. [43] proposed an indirect VSLAM system built upon a modified ORB-SLAM that enables high-accuracy localization and user interaction using the Occupancy Grid Mapping (OGM) method and a new 2D mapping module. Their system can reconstruct the environment map that shows the presence of a barrier as an equally spaced field of variables using the OGM, making it possible to navigate continuously and in real time while planning a route. The experimental examination of a generated dataset shows their approach functions in GPS-denied conditions. However, their technique struggles to function well in dynamic and complicated environments and has trouble appropriately matching the features in corridors and featureless conditions.

*CPA-SLAM*, a direct VSLAM method for RGB-D cameras that uses planes for tracking and graph optimization, was proposed by Ma et al. [123]. *Frame-to-keyframe* and *frame-to-plane* alignments are regularly integrated into their technique. They also introduced an image alignment algorithm for tracking the camera with respect to a reference keyframe and aligning the image with the planes. The keyframe data are used by *CPA-SLAM*, which looks for the closest short temporal and geographical distances to track. The system’s real-time performance of the tracking system was tested in with- and without-plane settings, with analyses performed on TUM RGB-D and ICL-NUIM datasets with indoor and outdoor scenes. However, it only supports a small number of geometric shapes, i.e., planes.

## 6. Current Trends

This section discusses what the current trends are and what is better to invest in the VSLAM domain according to the broad range of researched papers. These proposed systems cannot be recommended or rejected in this regard, as each is designed for a particular application and performs a specific objective. Thus, the authors concentrated on the shared aspects of the papers to extrapolate the hidden relationships between them and extract trendy topics. These aspects can include four main subjects: (1) the presence of a base framework to develop a system upon it, (2) the main target of the proposed solution, (3) the importance of semantic information for better performance, and (4) the evaluation criteria. The rest of this section introduces the statistics based on these four topics and analyzes the extracted outcomes.

### 6.1. Statistics

**Figure 4:**
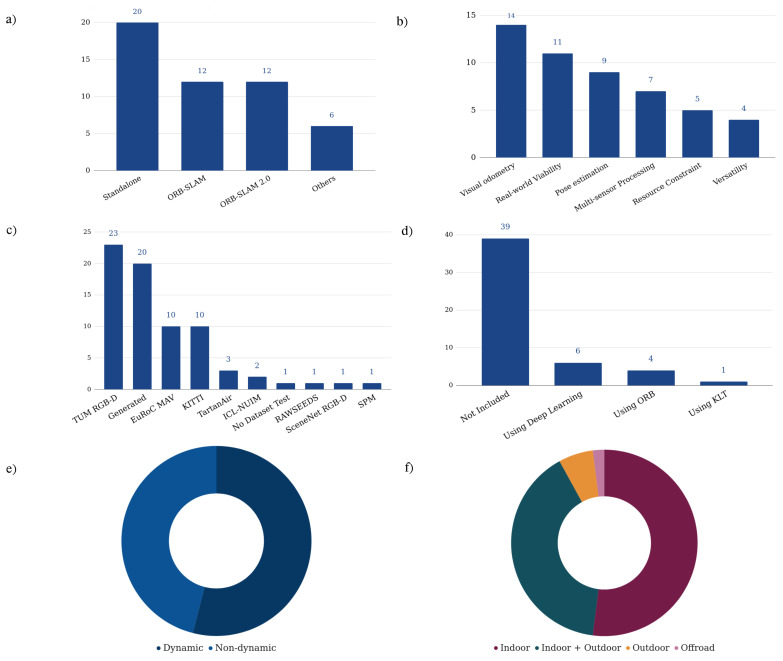
Analyzing the current trends of VSLAM approaches: (**a**) base SLAM system employed to implement a new approach, (**b**) the primary objective of the approach, (**c**) various datasets that the proposed methods were tested on, (**d**) the impact of utilizing semantic data in the proposed methods, (**e**) the amount of dynamic objects existing in the environment, (**f**) various types of environments the system tested on. Numbers updated due to adding five new papers.

Regarding the classification of the surveyed papers in the various aspects presented above, the processed data are visualized in Figure 4 to figure out the current trends in VSLAM. In Figure 4a, the majority of the proposed VSLAM systems are standalone applications that implement the whole procedure of localization and mapping using visual sensors from scratch. While ORB-SLAM 2.0 and ORB-SLAM are other *base* platforms employed to make a new framework, minimal approaches are based on other VSLAM systems, such as *PTAM* and *PoseSLAM*. Moreover, in terms of the objectives of VSLAM applications (**RQ2**), what tops the chart in Figure 4b is improving the *Visual Odometry* module. Thus, most of the recent VSLAMss are trying to resolve the problems of current algorithms in determining the position and orientation of the robots. *Real-world viability* and *pose estimation* are further fundamental objectives of proposing new VSLAM papers. Concerning the dataset used for evaluation in the surveyed papers (**RQ3**), Figure 4c illustrates that most works have been tested on the *TUM RGB-D* dataset. This dataset has been employed as the primary or one of the multiple baselines for evaluation in the reviewed manuscripts. Additionally, many researchers tend to perform experiments on the datasets *generated* by them. It is assumed that the primary motivation for generating a dataset is to show how a VSLAM method works in real-world scenarios and if it can be used as an end-to-end application. *EuRoC MAV* and *KITTI* are the next most popular datasets for evaluation in VSLAM works, respectively.

Another interesting piece of information extracted from Figure 4d concerns the impact of employing semantic data when using the VSLAM system. It can be seen that the majority of the reviewed papers do not include semantic data while processing the environment. The reason behind not utilizing semantic data could be due to the below reasons:

- The computational cost for training a model that recognizes objects and utilizing it for semantic segmentation is considerable in many cases, which might raise the processing time.
- The majority of the geometry-based VSLAM works are designed to work as plug-and-play devices so that they can employ camera data for localization and mapping with the least possible effort.
- Incorrect information extracted from the scene can also lead to adding more noise to the process.

When the environment is considered, it can be seen in Figure 4e, that more than half of the approaches can also work in dynamic environments with challenging conditions, while the remaining systems are only focused on an environment with no dynamic changes. Moreover, in Figure 4f, most of the approaches work in “indoor settings” or “both indoor and outdoor environments”, while the rest of the papers have only been tested in outdoor conditions. It should be mentioned that approaches that can only work in a particular circumstance with restricted assumptions might not produce the same accuracy if employed in other scenarios. That is one of the main reasons why some approaches only concentrate on a particular situation.

### 6.2. Analyzing the Current Trends

The current survey has reviewed the state-of-the-art visual SLAM approaches that have attracted massive attention and demonstrated their principal contributions to this field. Despite the wide range of reliable solutions and improvements in various modules of VSLAM systems over the past years, there are still many high-potential fields, and unsolved issues that are investigating them may lead to more robust approaches in the future evolution of SLAMs. In light of the wide range of visual SLAM approaches, the authors propose currently trending areas for investment and introduce the following open research directions:

#### 6.2.1. Various Feature Processing

Deep neural networks have shown encouraging results in various applications, including VSLAM [15], making them a significant trend in multiple fields of study. Due to their learning capabilities, these architectures have shown considerable potential to be utilized as reliable feature extractors to tackle different issues in VO and loop closure detection. CNNs can aid VSLAMs in precise object detection and semantic segmentation and can outperform traditional feature extraction and matching algorithms for correctly recognizing hand-crafted features. It has to be mentioned that since deep learning-based methods have been trained on datasets with large amounts of diversified data and limited object classes, there is always a risk of misclassification of dynamic points, causing false segmentation.

#### 6.2.2. Information Retrieval and Computational Cost Trade-Off

Generally, the processing cost and the quantity of information in the scene should always be balanced. In this perspective, dense maps allow VSLAM applications to record high-dimensional complete scene information, but doing so in real-time would be computationally demanding. Sparse representations, on the other hand, would fail to capture all the needed information due to their lower computational cost. It should also be noted that real-time performance is directly related to the camera’s frame rate, and frame losses in peak processing times can negatively affect the VSLAM system’s performance, regardless of algorithm performance. Moreover, VSLAMs typically take advantage of tightly coupled modules and modifying one module may adversely affect others, which makes the balancing task more challenging.

#### 6.2.3. Semantic Segmentation

Providing semantic information while creating the environment map can bring about beneficial information for the robot. Identifying objects in the camera’s field of view—e.g., doors, windows, people, etc.—is a trendy topic in current and future VSLAM works, as the semantic information can be used in pose estimation, trajectory planning, and loop closure detection modules. In this regard, 3D LiDAR-based frameworks, such as Situational Graph (S-Graph) [124], employ planar surfaces and semantic data to illustrate the surroundings face trouble in areas with a high presence of glass. With the widespread usage of object detection and tracking algorithms, semantic VSLAMs will undoubtedly be among the future solutions in this domain.

#### 6.2.4. Loop Closing Algorithms

One of the critical issues in any SLAM system is the drift problem and losing the feature tracks caused by the accumulated localization errors. Detection of drifts and loop closures to identify previously visited places contributes to high computation latency and cost in VSLAM systems [97]. The main reason is that the complexity of loop closure detection increases with the size of the reconstructed map. Moreover, combining the map data collected from various locations and refining the estimated poses are very complex tasks. With this, optimization and balancing of the loop closure detection module have massive potential for improvement. One of the common approaches for detecting loop closures is improving image retrieval by training a visual vocabulary based on local features and then aggregating them.

#### 6.2.5. Working in Challenging Scenarios

Working in a texture-less environment with few salient feature points often leads to drift errors in position and orientation in robots. As one of the primary challenges in VSLAM, this error may lead to system failure. Thus, considering complementary scene-understanding methods in feature-based approaches, such as object detection or line features, would be a trendy topic.

## 7. Conclusions

This paper presented a broad range of SLAM works equipped with visual sensors to collect data, known as visual SLAM (VSLAM). It investigated fifty state-of-the-art approaches and 10+ survey works published in the domain of VSLAM. The paper studied the evolution of VSLAM algorithms and discovered the highly matured frameworks that a majority of the further works are built upon them. Additionally, various setups concerning data acquisition means, visual features, target environments, technique evaluations, and semantic levels are addressed in this manuscript. The foremost contribution of this article compared to previous survey publications is categorizing various methodologies regarding their objectives in proposing new solutions. Moreover, recent VSLAM methods’ contemporary directions and well-known evaluation criteria concerning the test environment and benchmarks were other fundamental topics covered in this paper. The essay also introduced the existing research gaps and potential contributions that researchers can work on based on the current trendy issues in the VSLAM domain.

## Figures and Tables

**Table 1:** Commonly used datasets for VSLAM applications. *GT* in the table refers to the availability of ground-truth values.

Dataset Name	Year	Environment		Utilized Sensors									GT
		Indoor	Outdoor	GPS	LiDAR	IMU	Mono	Stereo	RGB-D	Event	Omni	UWB	
RAWSEEDS [51]	2006	✓	✓	✓	✓	✓		✓			✓		✓
KITTI [57]	2012		✓	✓	✓	✓	✓	✓					✓
ICL-NUIM [59]	2014	✓							✓				✓
TUM RGB-D [53]	2016	✓				✓			✓				✓
EuRoC MAV [55]	2016	✓				✓	✓	✓					✓
Event Camera [60]	2017		✓			✓				✓			✓
SceneNet RGB-D [52]	2017	✓							✓				✓
OpenLORIS [56]	2020	✓			✓	✓	✓	✓	✓				✓
TartanAir [58]	2020	✓	✓	✓			✓	✓	✓				✓
NTU VIRAL [54]	2021	✓	✓	✓		✓	✓					✓	✓

## Data Availability

Not applicable.

## References

[1] Khairuddin A.R., Talib M.S., Haron H. Review on simultaneous localization and mapping (SLAM). Proceedings of the 2015 IEEE International Conference on Control System, Computing and Engineering (ICCSCE).

[2] Vallivaara I., Haverinen J., Kemppainen A., Röning J. Magnetic field-based SLAM method for solving the localization problem in mobile robot floor-cleaning task. Proceedings of the 2011 15th International Conference on Advanced Robotics (ICAR).

[3] Zou Q., Sun Q., Chen L., Nie B., Li Q. (2021). A comparative analysis of LiDAR SLAM-based indoor navigation for autonomous vehicles. IEEE Trans. Intell. Transp. Syst..

[4] Geromichalos D., Azkarate M., Tsardoulias E., Gerdes L., Petrou L., Perez Del Pulgar C. (2020). SLAM for autonomous planetary rovers with global localization. J. Field Robot..

[5] Yang T., Li P., Zhang H., Li J., Li Z. (2018). Monocular vision SLAM-based UAV autonomous landing in emergencies and unknown environments. Electronics.

[6] Li J., Bi Y., Lan M., Qin H., Shan M., Lin F., Chen B.M. Real-time simultaneous localization and mapping for uav: A survey. Proceedings of the International Micro Air Vehicle Competition and Conference (IMAV).

[7] Liu Z., Chen H., Di H., Tao Y., Gong J., Xiong G., Qi J. Real-time 6d lidar slam in large scale natural terrains for ugv. Proceedings of the 2018 IEEE Intelligent Vehicles Symposium (IV).

[8] Gupta A., Fernando X. (2022). Simultaneous Localization and Mapping (SLAM) and Data Fusion in Unmanned Aerial Vehicles: Recent Advances and Challenges. Drones.

[9] Cadena C., Carlone L., Carrillo H., Latif Y., Scaramuzza D., Neira J., Reid I., Leonard J.J. (2016). Past, present, and future of simultaneous localization and mapping: Toward the robust-perception age. IEEE Trans. Robot..

[10] Zaffar M., Ehsan S., Stolkin R., Maier K.M. Sensors, slam and long-term autonomy: A review. Proceedings of the 2018 NASA/ESA Conference on Adaptive Hardware and Systems (AHS).

[11] Gao X., Zhang T. (2021). Introduction to Visual SLAM: From Theory to Practice.

[12] Filipenko M., Afanasyev I. Comparison of various slam systems for mobile robot in an indoor environment. Proceedings of the 2018 International Conference on Intelligent Systems (IS).

[13] Yeh Y.J., Lin H.Y. 3D reconstruction and visual SLAM of indoor scenes for augmented reality application. Proceedings of the 2018 IEEE 14th International Conference on Control and Automation (ICCA).

[14] Klein G., Murray D. Parallel tracking and mapping for small AR workspaces. Proceedings of the 2007 6th IEEE and ACM International Symposium on Mixed and Augmented Reality.

[15] Duan C., Junginger S., Huang J., Jin K., Thurow K. (2020). Deep Learning for Visual SLAM in Transportation Robotics: A Review. Transp. Saf. Environ..

[16] Outahar M., Moreau G., Normand J.M. (2021). Direct and Indirect vSLAM Fusion for Augmented Reality. J. Imaging.

[17] Macario Barros A., Michel M., Moline Y., Corre G., Carrel F. (2022). A Comprehensive Survey of Visual SLAM Algorithms. Robotics.

[18] Chen W., Shang G., Ji A., Zhou C., Wang X., Xu C., Li Z., Hu K. (2022). An Overview on Visual SLAM: From Tradition to Semantic. Remote Sens..

[19] Jia Y., Yan X., Xu Y. A Survey of simultaneous localization and mapping for robot. Proceedings of the 2019 IEEE 4th Advanced Information Technology, Electronic and Automation Control Conference (IAEAC).

[20] Kazerouni I.A., Fitzgerald L., Dooly G., Toal D. (2022). A Survey of State-of-the-Art on Visual SLAM. Expert Syst. Appl..

[21] Bavle H., Sanchez-Lopez J.L., Schmidt E.F., Voos H. (2021). From SLAM to Situational Awareness: Challenges and Survey. arXiv.

[22] Arshad S., Kim G.W. (2021). Role of deep learning in loop closure detection for visual and lidar slam: A survey. Sensors.

[23] Singandhupe A., La H.M. A review of slam techniques and security in autonomous driving. Proceedings of the 2019 Third IEEE International Conference on Robotic Computing (IRC).

[24] Saputra M.R.U., Markham A., Trigoni N. (2018). Visual SLAM and Structure from Motion in Dynamic Environments: A Survey. ACM Comput. Surv..

[25] Davison A.J., Reid I.D., Molton N.D., Stasse O. (2007). MonoSLAM: Real-time single camera SLAM. IEEE Trans. Pattern Anal. Mach. Intell..

[26] Ribeiro M.I. (2004). Kalman and extended kalman filters: Concept, derivation and properties. Inst. Syst. Robot..

[27] Viswanathan D.G. Features from accelerated segment test (fast). Proceedings of the 10th Workshop on Image Analysis for Multimedia Interactive Services.

[28] Newcombe R.A., Lovegrove S.J., Davison A.J. DTAM: Dense tracking and mapping in real-time. Proceedings of the 2011 International Conference on Computer Vision.

[29] Endres F., Hess J., Sturm J., Cremers D., Burgard W. (2013). 3-D mapping with an RGB-D camera. IEEE Trans. Robot..

[30] Salas-Moreno R.F., Newcombe R.A., Strasdat H., Kelly P.H., Davison A.J. Slam++: Simultaneous localisation and mapping at the level of objects. Proceedings of the 2013 IEEE Conference on Computer Vision and Pattern Recognition.

[31] Mendes E., Koch P., Lacroix S. ICP-based pose-graph SLAM. Proceedings of the 2016 IEEE International Symposium on Safety, Security, and Rescue Robotics (SSRR).

[32] Forster C., Pizzoli M., Scaramuzza D. SVO: Fast semi-direct monocular visual odometry. Proceedings of the 2014 IEEE International Conference on Robotics and Automation (ICRA).

[33] Engel J., Schöps T., Cremers D. LSD-SLAM: Large-scale direct monocular SLAM. Proceedings of the European Conference on Computer Vision.

[34] Mur-Artal R., Montiel J.M.M., Tardos J.D. (2015). ORB-SLAM: A versatile and accurate monocular SLAM system. IEEE Trans. Robot..

[35] Mur-Artal R., Tardós J.D. (2017). Orb-slam2: An open-source slam system for monocular, stereo, and rgb-d cameras. IEEE Trans. Robot..

[36] Campos C., Elvira R., Rodríguez J.J.G., Montiel J.M., Tardós J.D. (2021). Orb-slam3: An accurate open-source library for visual, visual–inertial, and multimap slam. IEEE Trans. Robot..

[37] Tateno K., Tombari F., Laina I., Navab N. Cnn-slam: Real-time dense monocular slam with learned depth prediction. Proceedings of the IEEE Conference on Computer Vision and Pattern Recognition.

[38] Engel J., Koltun V., Cremers D. (2017). Direct sparse odometry. IEEE Trans. Pattern Anal. Mach. Intell..

[39] He M., Zhu C., Huang Q., Ren B., Liu J. (2020). A review of monocular visual odometry. Vis. Comput..

[40] Gallego G., Delbrück T., Orchard G., Bartolozzi C., Taba B., Censi A., Leutenegger S., Davison A.J., Conradt J., Daniilidis K. (2020). Event-based vision: A survey. IEEE Trans. Pattern Anal. Mach. Intell..

[41] Jiao J., Huang H., Li L., He Z., Zhu Y., Liu M. Comparing representations in tracking for event camera-based slam. Proceedings of the IEEE/CVF Conference on Computer Vision and Pattern Recognition.

[42] Vidal A.R., Rebecq H., Horstschaefer T., Scaramuzza D. (2018). Ultimate SLAM? Combining events, images, and IMU for robust visual SLAM in HDR and high-speed scenarios. IEEE Robot. Autom. Lett..

[43] Xu L., Feng C., Kamat V.R., Menassa C.C. (2019). An occupancy grid mapping enhanced visual SLAM for real-time locating applications in indoor GPS-denied environments. Autom. Constr..

[44] Lowe D.G. (2004). Distinctive image features from scale-invariant keypoints. Int. J. Comput. Vis..

[45] Bay H., Tuytelaars T., Gool L.V. Surf: Speeded up robust features. Proceedings of the European Conference on Computer Vision.

[46] Calonder M., Lepetit V., Strecha C., Fua P. Brief: Binary robust independent elementary features. Proceedings of the European Conference on Computer Vision.

[47] Rublee E., Rabaud V., Konolige K., Bradski G. ORB: An efficient alternative to SIFT or SURF. Proceedings of the 2011 International Conference on Computer Vision.

[48] Karami E., Prasad S., Shehata M. (2017). Image matching using SIFT, SURF, BRIEF and ORB: Performance comparison for distorted images. arXiv.

[49] Fischler M.A., Bolles R.C. (1981). Random sample consensus: A paradigm for model fitting with applications to image analysis and automated cartography. Commun. ACM.

[50] Cui L., Ma C. (2019). SOF-SLAM: A semantic visual SLAM for dynamic environments. IEEE Access.

[51] Bonarini A., Burgard W., Fontana G., Matteucci M., Sorrenti D.G., Tardos J.D. Rawseeds: Robotics advancement through web-publishing of sensorial and elaborated extensive data sets. Proceedings of the 2006 International Conference on Intelligent Robots and Systems (IROS).

[52] McCormac J., Handa A., Leutenegger S., Davison A.J. Scenenet rgb-d: Can 5m synthetic images beat generic imagenet pre-training on indoor segmentation?. Proceedings of the IEEE International Conference on Computer Vision.

[53] Sturm J., Engelhard N., Endres F., Burgard W., Cremers D. A benchmark for the evaluation of RGB-D SLAM systems. Proceedings of the 2012 IEEE/RSJ International Conference on Intelligent Robots and Systems.

[54] Nguyen T.M., Yuan S., Cao M., Lyu Y., Nguyen T.H., Xie L. (2021). NTU VIRAL: A Visual-Inertial-Ranging-Lidar dataset, from an aerial vehicle viewpoint. Int. J. Robot. Res..

[55] Burri M., Nikolic J., Gohl P., Schneider T., Rehder J., Omari S., Achtelik M.W., Siegwart R. (2016). The EuRoC micro aerial vehicle datasets. Int. J. Robot. Res..

[56] Shi X., Li D., Zhao P., Tian Q., Tian Y., Long Q., Zhu C., Song J., Qiao F., Song L. Are we ready for service robots? the openloris-scene datasets for lifelong slam. Proceedings of the 2020 IEEE International Conference on Robotics and Automation (ICRA).

[57] Geiger A., Lenz P., Urtasun R. Are we ready for autonomous driving? the kitti vision benchmark suite. Proceedings of the 2012 IEEE Conference on Computer Vision and Pattern Recognition.

[58] Wang W., Zhu D., Wang X., Hu Y., Qiu Y., Wang C., Hu Y., Kapoor A., Scherer S. Tartanair: A dataset to push the limits of visual slam. Proceedings of the 2020 IEEE/RSJ International Conference on Intelligent Robots and Systems (IROS).

[59] Handa A., Whelan T., McDonald J., Davison A.J. A benchmark for RGB-D visual odometry, 3D reconstruction and SLAM. Proceedings of the 2014 IEEE International Conference on ROBOTICS and Automation (ICRA).

[60] Mueggler E., Rebecq H., Gallego G., Delbruck T., Scaramuzza D. (2017). The event-camera dataset and simulator: Event-based data for pose estimation, visual odometry, and SLAM. Int. J. Robot. Res..

[61] Yu C., Liu Z., Liu X.J., Xie F., Yang Y., Wei Q., Fei Q. DS-SLAM: A semantic visual SLAM towards dynamic environments. Proceedings of the 2018 IEEE/RSJ International Conference on Intelligent Robots and Systems (IROS).

[62] Wen S., Li P., Zhao Y., Zhang H., Sun F., Wang Z. (2021). Semantic visual SLAM in dynamic environment. Auton. Robot..

[63] Zou D., Tan P. (2012). Coslam: Collaborative visual slam in dynamic environments. IEEE Trans. Pattern Anal. Mach. Intell..

[64] Yang Y., Tang D., Wang D., Song W., Wang J., Fu M. (2020). Multi-camera visual SLAM for off-road navigation. Robot. Auton. Syst..

[65] Urban S., Hinz S. (2016). Multicol-slam-a modular real-time multi-camera slam system. arXiv.

[66] Zhu Y., Zheng C., Yuan C., Huang X., Hong X. Camvox: A low-cost and accurate lidar-assisted visual slam system. Proceedings of the 2021 IEEE International Conference on Robotics and Automation (ICRA).

[67] Nguyen T.M., Yuan S., Cao M., Nguyen T.H., Xie L. (2021). Viral slam: Tightly coupled camera-imu-uwb-lidar slam. arXiv.

[68] Rebecq H., Horstschaefer T., Scaramuzza D. Real-time visual-inertial odometry for event cameras using keyframe-based nonlinear optimization. Proceedings of the British Machine Vision Conference, University of Zurich.

[69] Nguyen T.H., Nguyen T.M., Xie L. (2020). Tightly-coupled ultra-wideband-aided monocular visual SLAM with degenerate anchor configurations. Auton. Robot..

[70] Zhou H., Zou D., Pei L., Ying R., Liu P., Yu W. (2015). StructSLAM: Visual SLAM with building structure lines. IEEE Trans. Veh. Technol..

[71] Pumarola A., Vakhitov A., Agudo A., Sanfeliu A., Moreno-Noguer F. PL-SLAM: Real-time monocular visual SLAM with points and lines. Proceedings of the 2017 IEEE International Conference on Robotics and Automation (ICRA), Marina Bay Sands.

[72] Gomez-Ojeda R., Moreno F.A., Zuniga-Noël D., Scaramuzza D., Gonzalez-Jimenez J. (2019). PL-SLAM: A stereo SLAM system through the combination of points and line segments. IEEE Trans. Robot..

[73] Lim H., Kim Y., Jung K., Hu S., Myung H. Avoiding degeneracy for monocular visual SLAM with point and line features. Proceedings of the 2021 IEEE International Conference on Robotics and Automation (ICRA).

[74] Bultmann S., Li K., Hanebeck U.D. Stereo visual slam based on unscented dual quaternion filtering. Proceedings of the 2019 22th International Conference on Information Fusion (FUSION).

[75] Munoz-Salinas R., Marin-Jimenez M.J., Medina-Carnicer R. (2019). SPM-SLAM: Simultaneous localization and mapping with squared planar markers. Pattern Recognit..

[76] Bruno H.M.S., Colombini E.L. (2021). LIFT-SLAM: A deep-learning feature-based monocular visual SLAM method. Neurocomputing.

[77] Naveed K., Anjum M.L., Hussain W., Lee D. (2022). Deep introspective SLAM: Deep reinforcement learning based approach to avoid tracking failure in visual SLAM. Auton. Robot..

[78] Peng Q., Xiang Z., Fan Y., Zhao T., Zhao X. (2022). RWT-SLAM: Robust Visual SLAM for Highly Weak-textured Environments. arXiv.

[79] Sun J., Shen Z., Wang Y., Bao H., Zhou X. LoFTR: Detector-free local feature matching with transformers. Proceedings of the IEEE/CVF Conference on Computer Vision and Pattern Recognition.

[80] Sun Y., Hu J., Yun J., Liu Y., Bai D., Liu X., Zhao G., Jiang G., Kong J., Chen B. (2022). Multi-objective Location and Mapping Based on Deep Learning and Visual Slam. Sensors.

[81] He K., Gkioxari G., Dollár P., Girshick R. Mask r-cnn. Proceedings of the 2012 IEEE International Conference on Computer Vision.

[82] Wojke N., Bewley A., Paulus D. Simple online and realtime tracking with a deep association metric. Proceedings of the 2017 IEEE International Conference on Image Processing (ICIP).

[83] Lin T.Y., Maire M., Belongie S., Hays J., Perona P., Ramanan D., Dollár P., Zitnick C.L. Microsoft coco: Common objects in context. Proceedings of the European Conference on Computer Vision.

[84] Badrinarayanan V., Kendall A., Cipolla R. (2017). Segnet: A deep convolutional encoder-decoder architecture for image segmentation. IEEE Trans. Pattern Anal. Mach. Intell..

[85] Cheng J., Sun Y., Meng M.Q.H. (2019). Improving monocular visual SLAM in dynamic environments: An optical-flow-based approach. Adv. Robot..

[86] Yang S., Zhao C., Wu Z., Wang Y., Wang G., Li D. (2022). Visual SLAM Based on Semantic Segmentation and Geometric Constraints for Dynamic Indoor Environments. IEEE Access.

[87] Li D., Shi X., Long Q., Liu S., Yang W., Wang F., Wei Q., Qiao F. DXSLAM: A robust and efficient visual SLAM system with deep features. Proceedings of the 2020 IEEE/RSJ International Conference on Intelligent Robots and Systems (IROS).

[88] Li G., Yu L., Fei S. (2021). A deep-learning real-time visual SLAM system based on multi-task feature extraction network and self-supervised feature points. Measurement.

[89] Steenbeek A., Nex F. (2022). CNN-Based Dense Monocular Visual SLAM for Real-Time UAV Exploration in Emergency Conditions. Drones.

[90] He K., Zhang X., Ren S., Sun J. Deep residual learning for image recognition. Proceedings of the 2016 IEEE Conference on Computer Vision and Pattern Recognition.

[91] Su P., Luo S., Huang X. (2022). Real-Time Dynamic SLAM Algorithm Based on Deep Learning. IEEE Access.

[92] Jocher G., Stoken A., Borovec J., Changyu L., Hogan A., Diaconu L., Ingham F., Poznanski J., Fang J., Yu L. (2020). ultralytics/yolov5: V3.1—Bug Fixes and Performance Improvements. https://zenodo.org/record/4154370#.Y4LNkHZBxPY.

[93] Chen J., Xie F., Huang L., Yang J., Liu X., Shi J. (2022). A Robot Pose Estimation Optimized Visual SLAM Algorithm Based on CO-HDC Instance Segmentation Network for Dynamic Scenes. Remote Sens..

[94] Muñoz-Salinas R., Medina-Carnicer R. (2020). UcoSLAM: Simultaneous localization and mapping by fusion of keypoints and squared planar markers. Pattern Recognit..

[95] Liu G., Zeng W., Feng B., Xu F. (2019). DMS-SLAM: A general visual SLAM system for dynamic scenes with multiple sensors. Sensors.

[96] Bian J., Lin W.Y., Matsushita Y., Yeung S.K., Nguyen T.D., Cheng M.M. Gms: Grid-based motion statistics for fast, ultra-robust feature correspondence. Proceedings of the IEEE Conference on Computer Vision and Pattern Recognition.

[97] Xu J., Cao H., Li D., Huang K., Qian C., Shangguan L., Yang Z. Edge assisted mobile semantic visual slam. Proceedings of the IEEE INFOCOM 2020-IEEE Conference on Computer Communications.

[98] Schlegel D., Colosi M., Grisetti G. Proslam: Graph SLAM from a programmer’s Perspective. Proceedings of the 2018 IEEE International Conference on Robotics and Automation (ICRA).

[99] Bavle H., De La Puente P., How J.P., Campoy P. (2020). VPS-SLAM: Visual planar semantic SLAM for aerial robotic systems. IEEE Access.

[100] Redmon J., Farhadi A. YOLO9000: Better, faster, stronger. Proceedings of the 2017 IEEE Conference on Computer Vision and Pattern Recognition.

[101] Tseng P.Y., Lin J.J., Chan Y.C., Chen A.Y. (2022). Real-time indoor localization with visual SLAM for in-building emergency response. Autom. Constr..

[102] Sumikura S., Shibuya M., Sakurada K. Openvslam: A versatile visual slam framework. Proceedings of the 27th ACM International Conference on Multimedia.

[103] Ben Ali A.J., Hashemifar Z.S., Dantu K. Edge-SLAM: Edge-assisted visual simultaneous localization and mapping. Proceedings of the 18th International Conference on Mobile Systems, Applications, and Services.

[104] Ferrera M., Eudes A., Moras J., Sanfourche M., Le Besnerais G. (2021). OV^2^SLAM: A Fully Online and Versatile Visual SLAM for Real-Time Applications. IEEE Robot. Autom. Lett..

[105] Teed Z., Deng J. (2021). Droid-slam: Deep visual slam for monocular, stereo, and rgb-d cameras. Adv. Neural Inf. Process. Syst..

[106] Bonetto E., Goldschmid P., Pabst M., Black M.J., Ahmad A. (2022). iRotate: Active Visual SLAM for Omnidirectional Robots. Robot. Auton. Syst..

[107] Xiao L., Wang J., Qiu X., Rong Z., Zou X. (2019). Dynamic-SLAM: Semantic monocular visual localization and mapping based on deep learning in dynamic environment. Robot. Auton. Syst..

[108] Bloesch M., Czarnowski J., Clark R., Leutenegger S., Davison A.J. CodeSLAM—learning a compact, optimisable representation for dense visual SLAM. Proceedings of the 2018 IEEE Conference on Computer Vision and Pattern Recognition.

[109] Wang S., Clark R., Wen H., Trigoni N. Deepvo: Towards end-to-end visual odometry with deep recurrent convolutional neural networks. Proceedings of the 2017 IEEE International Conference on Robotics and Automation (ICRA), Marina Bay Sands.

[110] Parisotto E., Singh Chaplot D., Zhang J., Salakhutdinov R. Global pose estimation with an attention-based recurrent network. Proceedings of the IEEE Conference on Computer Vision and Pattern Recognition Workshops.

[111] Czarnowski J., Laidlow T., Clark R., Davison A.J. (2020). Deepfactors: Real-time probabilistic dense monocular slam. IEEE Robot. Autom. Lett..

[112] Dai A., Chang A.X., Savva M., Halber M., Funkhouser T., Nießner M. Scannet: Richly-annotated 3d reconstructions of indoor scenes. Proceedings of the IEEE Conference on Computer Vision and Pattern Recognition.

[113] Dai X.Y., Meng Q.H., Zheng W.J., Zhu S.K. Monocular Visual SLAM based on VGG Feature Point Extraction. Proceedings of the 2020 39th Chinese Control Conference (CCC).

[114] Simonyan K., Zisserman A. (2014). Very deep convolutional networks for large-scale image recognition. arXiv.

[115] Gu X., Wang Y., Ma T. DBLD-SLAM: A Deep-Learning Visual SLAM System Based on Deep Binary Local Descriptor. Proceedings of the 2021 International Conference on Control, Automation and Information Sciences (ICCAIS).

[116] Balntas V., Lenc K., Vedaldi A., Mikolajczyk K. HPatches: A benchmark and evaluation of handcrafted and learned local descriptors. Proceedings of the 2017 IEEE Conference on Computer Vision and Pattern Recognition (CVPR).

[117] Kerl C., Sturm J., Cremers D. Dense visual SLAM for RGB-D cameras. Proceedings of the 2013 IEEE/RSJ International Conference on Intelligent Robots and Systems.

[118] Kerl C., Sturm J., Cremers D. Robust Odometry Estimation for RGB-D Cameras. Proceedings of the 2013 IEEE International Conference on Robotics and Automation (ICRA).

[119] Ila V., Porta J.M., Andrade-Cetto J. (2009). Information-based compact pose SLAM. IEEE Trans. Robot..

[120] Li A., Wang J., Xu M., Chen Z. (2021). DP-SLAM: A visual SLAM with moving probability towards dynamic environments. Inf. Sci..

[121] Dong E., Xu J., Wu C., Liu Y., Yang Z. Pair-navi: Peer-to-peer indoor navigation with mobile visual slam. Proceedings of the IEEE INFOCOM 2019-IEEE Conference on Computer Communications.

[122] Li B., Zou D., Sartori D., Pei L., Yu W. Textslam: Visual slam with planar text features. Proceedings of the 2020 IEEE International Conference on Robotics and Automation (ICRA).

[123] Ma L., Kerl C., Stückler J., Cremers D. CPA-SLAM: Consistent plane-model alignment for direct RGB-D SLAM. Proceedings of the 2016 IEEE International Conference on Robotics and Automation (ICRA).

[124] Bavle H., Sanchez-Lopez J.L., Shaheer M., Civera J., Voos H. (2022). Situational Graphs for Robot Navigation in Structured Indoor Environments. arXiv.

